# COVID-19 and migrants: lessons for pandemic preparedness from the Malaysian experience

**DOI:** 10.1186/s12992-023-00988-9

**Published:** 2023-11-27

**Authors:** Sharuna Verghis

**Affiliations:** Jeffery Cheah School of Medicine and Health Sciences, Monash University Malaysia, Jalan Lagoon, Selatan Bandar Sunway, Subang Jaya, 47500 Malaysia

**Keywords:** Migration, Migrant workers, Refugees, COVID-19, Pandemic, Malaysia, Migrant health policy, Health equity, Health in All Policies, Pandemic preparedness

## Abstract

**Background:**

Aligning with global evidence related to migrants and COVID-19, the pandemic highlighted and exposed long-standing structural inequities in the context of migrant populations in Malaysia who experienced a disproportionate level of exposure to SARS-CoV-2 and COVID-19 morbidity, as well as exacerbated precarity during COVID-19 owing to disruptions to their livelihoods, health, and life.

**Main body:**

Focusing on COVID-19 and migrant workers in Malaysia, this review addresses two research queries: (i) what are the policy responses of the government toward migrants with regard to COVID-19? (ii) what are the lessons learned from the Malaysian experience of COVID-19 and migrants that can inform pandemic preparedness, especially regarding migrant health policy?

The review used Arksey and O’Malley’s methodological framework refined by Levac, Colquhoun, and O’Brien. In addition to the PubMed, Web of Science, Scopus, and EBSCO databases, and Malaysian English language newspapers, including the Malay Mail, Malaysiakini, and the New Straits Times, the search also included reports from the websites of government ministries and departments, such as the Immigration Department, Ministry of Human Resources, Ministry of Health, and the International Trade and Industry Ministry.

**Conclusion:**

Using the case example of Malaysia and the policy approach toward migrant populations in Malaysia during the height of the COVID pandemic in 2020 and 2021, this paper unravels complex pathways and inter-linkages between the contexts of migration and health which coalesced to engender and exacerbate vulnerability to disease and ill-health for the migrant workers. The lack of coordination and coherence in policies addressing migrant workers during the pandemic, the normalization of cheap and disposable labor in neoliberal economic regimes, and the securitization of migration were key factors contributing to the failure of migration policies to provide protection to migrant workers during COVID-19. The review suggests that policy approaches embodying the principles of Health in All Policies, a whole-of-society approach, and the promotion of safe, just, and regular migration, predicated on equity and inclusion, are integral to a comprehensive and effective response to pandemics such as COVID-19.

## Introduction

In line with the global evidence on migrant populations and COVID-19, the pandemic brought to light longstanding structural inequities within the context of migrant populations in Malaysia. These populations were disproportionately exposed to SARS-CoV-2 and COVID-19 morbidity during the pandemic. Additionally, they faced increased vulnerability due to disruptions in their livelihoods, health, and overall well-being.

This review centres on COVID-19 and migrant workers in Malaysia to examine the government’s policy responses to this specific demographic during the pandemic. By drawing insights from the Malaysian experience with COVID-19 and migrants, the review seeks to extract lessons that can contribute to enhanced pandemic preparedness, particularly related to migrant health policy.

In the context of future pandemic preparedness, the Malaysian experience serves as a notable lesson applicable to nations with sizable migrant populations, It supplements the extant literature, emphasising how COVID-19 exposed pre-existing structural inequities within migrant populations [1–3], including migrant related policies, which exacerbated their precarity and risks of acquiring COVID-19. Within the evolving landscape of research in Malaysia, which has primarily focused on the pandemic’s broader impact on migrants [3–10], this study seeks to enhance the understanding of the pandemic’s impact on migrants. It does so by focusing on the role of policy in the development of precarity and vulnerability within this population during the pandemic. Crucially, the study emphasises the insufficiency of the Ministry of Health’s progressive COVID-19 healthcare accessibility and vaccine equity policies in mitigating virus transmission (challenges faced by migrants outside Malaysia) when broader non-health policies are not conceptually aligned with equity-promoting health policies.

## Background: migrant populations in Malaysia

### The context of migration in Malaysia

Propelled by globalisation and labour market forces, Malaysia’s economic development has relied heavily on foreign migrant labour [11]. A mixed migration dynamic characterises the migration context in Malaysia. Mixed migration is defined as complex migratory population movements that include refugees, asylum-seekers, economic migrants, unaccompanied minors, environmental migrants, smuggled persons, victims of trafficking and stranded migrants, among others, as opposed to migratory population movements that consist entirely of one category of migrants [12, 13]. Mixed migration flows are characterised by the irregular nature of the movement of people, diverse factors leading to the movement of people, and the differentiated needs and profiles of the persons involved [12]. In this context, Malaysia is host to one of the largest migrant populations in Asia and is one of the top destinations for low-skilled migrant labour. By 2022, the Malaysian Immigration Department registered about 1.4 million migrant workers [14], who comprise 15% of the total employed persons in the country [15]. Many estimates suggest that, including undocumented migrants, there are an estimated three million [16] and even possibly 5.5 million migrants in Malaysia [17]. As a mixed migration setting, the country is also a major destination for forced migrant populations like refugees, asylum seekers, and stateless persons, regarded as undocumented migrants, because Malaysia does not have a legal or administrative framework for refugees. By the end of August 2022, about 185,920 refugees and asylum-seekers were registered with the United Nations High Commissioner for Refugees (UNHCR) in Malaysia, of whom 159,190 (largely Rohingya) are from Myanmar, and the rest belong to about 40 different countries [18]. Refugees and asylum seekers, similar to other undocumented migrants, work in the exploitative informal sector [19]. Nevertheless, refugees registered with UNHCR, despite their undocumented status, do have some pre-existing limited benefits. These include reduced user fees in public hospitals and protection against repatriation to their home country if apprehended due to the absence of valid documentation.

Malaysia lacks a comprehensive migration policy framework, and this leads to various levels of insecurity and vulnerability for both documented and undocumented migrants. Documented migrant workers, often comprising semi-skilled and unskilled workers, are reported to experience precarity due to circular labor migration policies defined as the repeated temporary movement of persons between destination and countries of origin [20]. This approach is reflected in practices that include short, fixed-term employment contracts, restrictions on transferring work permits to different sectors or employers, and the inconsistent implementation of immigration control policies, marked by periodic mass raids, arrests, and amnesty programs [21]. Refugees and asylum seekers who are considered undocumented are prohibited from formal work, and their children cannot attend public schools. Non-citizens, including both documented and undocumented migrants, are subject to significantly higher user fees in public healthcare facilities compared to citizens. These fees can be 35–40 times greater than what citizens pay for hospital ward services [22]. Notably, refugees and asylum seekers receive a 50% discount on these fees in public healthcare facilities, which are still unaffordable [23]. It is important to highlight that hospitals are required to report undocumented migrants to law enforcement authorities [24], although this is not consistently practiced.

Malaysia is state party to only two of the core international human rights treaties: the Convention on the Rights of the Child (CRC), 1989 and the Convention on the Elimination All forms of Discrimination against Women (CEDAW), 1979, besides several International Labour Organisation (ILO) conventions such as the ILO Forced Labour Convention (No. 29); Right to Organise and Collective Bargaining Convention (No. 98), Equal Remuneration Convention (No.100), and the Employment Service Convention (No. 88) which do not differentiate on the basis of citizenship. It has not ratified the 1951 Refugee Convention and its 1967 Protocol or the International Convention on the Protection of the Rights of All Migrant Workers and Members of Their Families (ICMW), 2007. The CEDAW Committee in its Concluding Observations in 2018 raised concerns about problems in access to healthcare, arrest and detention of migrant women, and the denial of equal labour rights to domestic workers who are mostly migrants [25]. Malaysia has also endorsed the Global Compact for Safe, Orderly and Regular Migration; however, the voluntary nature of subscription to an ambiguous global norm does not ensure the protection of migrant worker rights.

### Exacerbated precarity of migrants during COVID-19

During the COVID-19 pandemic, migrant communities in Malaysia faced significant challenges, suffering, and disruptions to their livelihoods, health, and even lives. Thousands lost their jobs. The Human Resources Ministry advised that ‘if a lay-off is inevitable, foreign employees should be terminated first’ [26]. Numerous instances of labor rights violations and the exploitation of migrant workers were reported during the COVID-19 pandemic [27, 28].

Daily-wage migrant workers, both documented and undocumented, faced particularly challenging conditions, including food insecurity and limited access to healthcare [29]. Undocumented migrants, in particular, had barriers to accessing healthcare due to raids, Movement Control Orders (MCO), and financial constraints, as exemplified by the case of an undocumented woman who gave birth at home due to fear of arrest [30]. Stranded as the government monitored entry and exit points to prevent large gatherings of migrants who might be considering clandestine exits [31], and barricaded their accommodations with barbed wire which were patrolled by security forces [32], migrants relied on patchy and intermittent assistance from civil society and individuals [33].

Migrant populations also lacked information about COVID-19 in their native languages from official sources, leading to misconceptions about the pandemic and protective measures [34].

### Vulnerability of migrant communities to COVID-19

Regarding COVID-19 infection among migrants, one estimate indicated that as of August 2020, migrants comprised one-third of the total COVID-19 cases in the country [35]. According to the Ministry of Health (MOH) data, by December 9, 2021, foreigners accounted for 15.9% of Malaysia’s approximately 2.67 million cumulative confirmed COVID-19 cases [36]. However, it is noteworthy that by the end of December 2021, publicly available data on the official GitHub account of the Ministry of Health [37] did not provide disaggregated data on COVID-19 cases among migrant populations.

The absence of disaggregated health and health utilization data for migrant populations has been an ongoing issue in Malaysia, even prior to the pandemic. This lack of data has hindered the development of appropriate policies, programs, and effective advocacy efforts for migrant health. Despite these data gaps, reports have linked some of the larger workplace and detention center COVID-19 clusters to migrant populations [3].

However, COVID-19 clusters began emerging in immigration detention centres. The outbreaks in overcrowded detention centres were attributed to the Ministry of Home Affairs’ securitized approach to pandemic control [38] leading to mass arrests and raids with the intention of preventing the movement of migrants and the potential spread of the disease [39]. The Copenhagen School which redefined the concept of securitization, characterises the process as a speech-act through which an ‘issue is presented as an existential threat, requiring emergency measures and justifying actions outside the normal bounds of political procedure’ [40] p.24. This approach contrasted the inclusive COVID-19 approach of the Ministry of Health to COVID-19 testing and treatment. Additionally, the securitized approach led to the implementation of concerning practices targeted at migrants. According to media reports, after they were apprehended by authorities, migrants were subjected to mass disinfection exercises, with disinfectant sprayed directly on their heads and hands as a preventive measure against COVID-19. Health experts criticized this practice as demeaning and unnecessary [41].

The initial workplace cluster, the I.N. Cluster, was identified on March 24, 2020, involving two migrant workers [37]. The Ministry of Health’s GitHub database recorded workplace clusters from May 2020, with a significant surge from October 2020 onward [37]. Notably, early data indicated that the majority of positive cases at workplaces in early 2020 were migrants [37], including individuals from Bangladesh, Nepal, Indonesia, Myanmar, India, Cambodia, and Vietnam. The sectors most affected were manufacturing (780 clusters), the service sector (260 clusters), and construction (181 clusters). The states of Selangor, Johor, and Kuala Lumpur, known for their high industrialization and substantial migrant worker populations [42], recorded the highest number of workplace clusters [43].

In addition, trade union leaders reported unreported cases, including migrant workers who died of COVID-19 without seeking healthcare, often due to undocumented status or lack of employer-provided healthcare [44]. In August 2021, when Brought in Dead (BID) cases accounted for a significant portion of fatalities, the high number of migrant BID cases raised concerns about limited healthcare access and greater exposure to risk [45]. BID refers to COVID-19 deaths occurring outside of the hospital and later brought to the hospital, and fulfilling the criteria of a confirmed COVID-19 diagnosis through laboratory tests (e.g., Gene Expert), having an epidemiological link with other COVID-19 cases, or having supportive radiological investigation results suggestive of COVID-19 infection [46]. A study investigating the effects of COVID-19 on on inpatient deaths and brought-in-dead cases in Malaysia from March 17, 2020, to November 3, 2021, identified a higher likelihood of brought-in-dead (BID) cases linked to non-Malaysian individuals who comprised roughly one-third (31.9%) of the total BID [47].

As shown in the upcoming section on COVID-19 policies for migrant workers, immigration, labor, and housing policies, along with their enforcement or lack thereof, played a major role in worsening the risk, infections, and difficulties faced by migrant communities, even though health policies were equitable.

### Definitions

For this review on the policy responses of the government towards migrants, ‘migrants’ only include a labor migrant who is ‘engaged or has been engaged in a remunerated activity in a state of which he or she is not a citizen’ (Art 2.1) [48]. Refugees, asylum seekers, and stateless individuals are excluded from this review due to its policy-oriented scope, which centers on the formal recognition of labour migrants and the lack of recognition for refugees within the legal and administrative frameworks of the country. Moreover, the legal ambiguity surrounding UNHCR-registered refugees who benefit from subsidized healthcare and relative protection from repatriation to their home country differentiates their experiences from those of undocumented labour migrants. Additionally, there is a distinction between undocumented migrants, who may access consular services but lack welfare protection provided by their embassies, and refugees and asylum seekers who benefit from the active engagement of UNHCR with the Malaysian government for their protection needs. Thus, despite the interesting exploration that the liminal state of refugees and asylum seekers offers in understanding the concept of undocumented status and its associated legal protections, this review has excluded refugees due to the absence of pandemic-related migration policies specifically addressing this demographic in terms of both intent and content.

The above definition of migrant workers in Malaysia encompasses low and semi-skilled workers primarily originating from Bangladesh, Indonesia, Nepal, Myanmar, and India [14]. The majority of migrant workers in Malaysia are primarily employed in manufacturing (36.9%), construction (21.7%), plantation (11.9%), and services (15.2%) sectors and sub-sectors, with about eight % comprising domestic workers [14]. It is noteworthy that as of June 1, 2021, domestic workers, who are predominantly women, have been brought under the purview of the Employees’ Social Security Act (Act 4) and the Employment Insurance System (Act 800). Nonetheless, despite their inclusion under the Employment Act of 1955, legal entitlements pertaining to paid leave, rest days, working hours, break hours, and maternity protection do not apply to this group [49].

Consistent with the overarching emphasis on policy influence on migrants in Malaysia during the pandemic, this scoping review addresses the following specific research questions:

1. What are the policy responses of the government toward migrants with regard to COVID-19?
2. What are the lessons learned from the Malaysian experience of COVID-19 and migrants that can inform pandemic preparedness, especially regarding migrant health policy?

## Methods

The scoping study framework developed by Arksey and O’Malley [50] and refined by Levac, Colquhoun, and O’Brien [51] was deemed most suitable since the aim was to summarize and disseminate findings on the topic to policymakers and migration and migration health practitioners and undertake a preliminary mapping of issues for future research on migrant health and pandemic preparedness.

This scoping study framework revolves around six steps, (1) identifying the research question; (2) identifying relevant articles; (3) selecting articles; (4) charting the data; (5) collating, summarizing, and reporting the results; and (6) consultation, the final optional step, which has been omitted in this review. While Arksey and O’Malley [50] suggest that consultation, which offers opportunities to gather additional references and insights beyond the existing literature through stakeholder engagement is optional, Levac et al. [51] underscore the significance of clearly defining the purpose of this step. The omission of this step was significantly influenced by the substantial limitations and resource constraints imposed by COVID-19. Further to this limitation, the decision to regard this step as a post-review knowledge translation activity [52] was guided by the broad and exploratory character of the research questions, which necessitated an iterative approach, and the review’s overarching aim which is the eventual dissemination of findings to policymakers and practitioners.

This choice of a scoping study to explore this topic was also supported by the scarce [53], yet heterogeneous literature that had to be reviewed [54], which required an iterative search and retrieval process.

### Search strategy

A literature search encompassing English-language studies published between February 2020 and January 2022 was conducted across multiple databases, including PubMed, Web of Science, Scopus, and EBSCO. The search was confined to articles published in English. Employing a Boolean search strategy, the query combined the terms “Malaysia” AND “COVID” OR “COVID-19” OR “pandemic” AND “migrants” OR “foreign workers” OR “undocumented migrants” OR “illegal migrants.” This search yielded a total of 99 articles initially. Upon the elimination of duplicates, the search yielded 42 unique papers, which were subsequently subjected to screening for relevance based on title and abstract content. After this initial screening of titles and abstracts, 11 papers were excluded. The exclusion criteria included papers that did not address migrant workers or Malaysia or did not focus on the intersection of COVID-19 and migrant populations. Any paper that addressed migrant workers in Malaysia during COVID-19 to any extent was included in the study. After a thorough full-text review, a total of 19 articles were chosen for inclusion in the study. Among the 19 selected articles, four addressed both migrant workers and refugees. For the purpose of this review, pertinent information concerning migrant workers was extracted from these four papers during the full-text review.

Owing to the inadequacy of papers on COVID-19 policies related to migrants, the search also encompassed English-language newspapers from sources such as the Malay Mail, Malaysiakini, and the New Straits Times, published within the period from February 2020 to January 2022. These newspapers were selected to capture information regarding government policies concerning COVID-19 and migrant workers, as well as the pandemic’s impact on this demographic. Papers unrelated to migrant workers in the context of COVID-19 or its repercussions were systematically excluded. This approach resulted in the retrieval of 107 relevant articles from a pool of 637 news articles, aligning with the predefined inclusion criteria. These retrieved articles not only met the inclusion criteria but also contributed to addressing the research question concerning the COVID-19 policies implemented by the Malaysian government regarding migrant populations.

Additionally, a comprehensive search was conducted on government ministry and department websites, including those of the Immigration Department, Ministry of Human Resources, Ministry of Health, and the International Trade and Industry Ministry, to gather pertinent information. Furthermore, to augment the research, additional articles and papers were hand-searched through citations within both published and grey literature sources.

## Analysis

Thematic analysis was employed for data analysis following the six-step approach proposed by Braun and Clarke [55]. The initial step involved familiarizing with the data through repeated reading and note-taking, facilitating the creation of a timeline detailing events related to government policy actions directed towards migrant workers during the study period. Subsequently, in the second step, the content of each government policy and its implications for migrant workers were extracted and summarized, aligning with the research questions. Moving to the third step, open coding was used to generate codes deductively from the data, guided by the research questions’ focus on identifying the government’s policy responses to migrants during the COVID-19 pandemic. Based on this, themes emerged and were reviewed in relation to the coded extracts and the entire dataset. Finally, the themes were defined and named to encapsulate the overarching narrative evident in the data, culminating in the composition of the analysis.

This review sought to identify the government’s policy responses toward migrants with regard to COVID-19 and lessons that could be learned for future pandemic preparedness, especially in terms of migrant health policy. The government’s policy and legislative responses directed toward migrant workers were categorized into the following three overarching themes:

1. Incoherence in migrant worker policy and gaps in inter-ministerial coordination: This theme revolves around the absence of a coherent policy framework and the lack of coordination among various ministries during COVID-19. These factors resulted in vulnerabilities and fear among migrant workers, uncertainty among their employers, and hindered the Ministry of Health’s efforts to control the pandemic.
2. Normalization of cheap and disposable migrant labor in neoliberal economic regimes: This theme highlights the significance of cheap and disposable migrant labor within pre-existing neoliberal economic structures. It underscores how these labor practices, prevalent before COVID-19, became normalized during the humanitarian crisis of the pandemic.
3. Securitization of migration in the context of public health policies: This theme emphasizes the strengthening of migration securitization within the framework of public health policies designed to curb COVID-19 transmission. This approach led to the widespread arrest and detention of migrants, the State’s absence of action regarding xenophobic hate speech directed at migrants, and the emergence of avoidable COVID-19 clusters in detention centres.

### Incoherence in migrant worker policy and gaps in inter-ministerial coordination 

As illustrated in Table 1 and this section, the examination of the government’s policy responses toward migrant workers reveals an inconsistent and poorly coordinated approach marked by divergent policies across different ministries and agencies, each operating under distinct conceptual frameworks and objectives, in their efforts to address the pandemic.

**Table 1:** Timeline of COVID-19 policies and legislation related to migrant workers

Timeline	Immigration/home affairs policy	Health policy	Labor policy	Social protection policy
2020 February	The then Senior Minister Datuk Seri Ismail Sabri Yaakob gave assurances that undocumented migrants should not be worried about their status as the government has agreed not to make it an issue but would instead focus on curbing the pandemic	The first detection of COVID-19 in the migrant population in Malaysia in the international *Tabligh* (religious gathering)		
2020 March	Prime Minister Tan Sri Muhyiddin Yassin initially stated that foreigners would have to pay for COVID-19 tests and treatment at government hospitals. This approach was likely in keeping with the healthcare financing policy to charge unsubsidized fees to foreigners in government hospitals	The Ministry of Health later clarified that COVID-19 tests and treatment at government hospitals would be free for foreigners	The Human Resources Ministry advised that ‘if a lay-off is inevitable, foreign employees should be terminated first’	
2020 April/May	Beginning of mass arrests and detention of migrants with the objective of stopping the transmission of the COVID-19 virus Security at land, sea and air borders are tightened by the Police, Army, Malaysian Maritime Enforcement Agency, Immigration Department, People’s Volunteers Corps (RELA) Beginning of online xenophobic and hate speech against migrants. Lack of action by the government to stop it Selangor Mansion, Malayan Mansion and Menara City One which house migrant workers is barricaded with barbed wire and put under Enhanced Movement Control Order (EMCO). The objective was to reduce the transmission of the virus	The Director-General of Health warned against discriminating against migrants in healthcare	Reports of overcrowded lodgings of migrants emerge and keep getting attention till early 2021 Calls increase to enforce the Workers’ Minimum Standards of Housing and Amenities (Amendment) Act 2019 (Act 446) which was passed in July 2019 and gazetted in September 2019 Act 446 sought to improve housing conditions for migrant workers by imposing statutory obligations on employers regarding migrant worker accommodation. However, its enforcement was stymied by lack of enforcement	
2020 May		The beginning of outbreaks of COVID-19 clusters in the immigration detention centers. The Ministry of Health initiates systematic mass screening and decontamination procedures at the immigration detention centers and arranges quarantine and treatment, after which undocumented migrants would be deported upon completing treatment	The government agreed that employers of documented migrants would be subsidized. The Social Security Organisation (SOCSO) to which migrant workers make monthly contributions would bear the cost of COVID-19 screening for documented foreign workers who make SOCSO contributions. The objective was to ease the burden of the employers who would have otherwise had to fork out the cost of routine workplace base testing for COVID-19	
2020 August				The government announces social protection programs for Malaysians. Migrant workers are excluded
2020 November	The government announced the Return Recalibration Program. This was to facilitate voluntary return of migrant workers who were undocumented before the pandemic began The Home Minister announced employers in the construction, manufacturing, plantation and agriculture sectors will be allowed to legally employ undocumented foreign workers under the Labour Recalibration Plan		The government announced Labor Recalibration Program, which sought to provide a pathway to employment for migrant workers who lost their jobs because of COVID-19 related layoffs. It was not successful	
2020 December			Investigative reports by journalists reveal a lack of enforcement of workplace policies instituted to reduce COVID-19 transmission in the glove manufacturing sector	
2021 February		Malaysia extends free COVID-19 vaccination to migrants	The Emergency (Employees’ Minimum Standards of Housing, Accommodations, and Amenities (Amendment) Ordinance 2021 (Act 446) came into effect; however, enforcement was postponed giving employers more time	
2021 March			U.S. Customs and Border Protection (CBP) begins seizing disposable gloves produced in Malaysia under conditions of forced labor, reflecting inadequate enforcement of workplace protection	
2021 November			The Labor Minister clarified that the Recalibration programs were not meant to legalize foreign workers	

Initially, Prime Minister Tan Sri Muhyiddin Yassin indicated that foreigners would be responsible for the costs of COVID-19 tests and treatment at government hospitals. However, aligning with a more equitable public health approach, the Ministry of Health later clarified that these services would be provided for free [56]. Regarding screening, the government agreed to subsidize employers for COVID-19 screening for documented foreign workers contributing to the Social Security Organisation (SOCSO) [57]. Nonetheless, starting in April 2020, when undocumented migrants hesitated to come forward for testing and treatment, the Ministry of Health reassured all migrant workers that they could access free COVID-19 care without fear of arrest. Contrary to this, the Immigration Department and the Ministry of Home Affairs initiated mass raids, arrests, and detention [58–60]. This reversal from their March 2020 stance not to arrest undocumented migrants resulted in avoidable COVID-19 clusters in overcrowded immigration detention centres [61].

A similar situation unfolded in 2021 when Health Minister Khairy Jamaluddin announced that undocumented migrants could freely get vaccinated. However, the Home Minister subsequently reversed this stance, citing the need to safeguard citizens and migrants from a new wave of the virus [59]. This flip-flopping contributed to fear of arrest and vaccine hesitancy among undocumented migrants [62].

Likewise, the Recalibration Programs [63, 64] which aimed at facilitating the return of undocumented individuals pre-COVID-19 (Repatriation Recalibration Plan) and regularizing those who became undocumented due to pandemic-related layoffs (Labor Recalibration Plan) also yielded mixed messages and proved ineffective. Initially, in 2020, the immigration department announced that details regarding the Repatriation Recalibration Plan and the Labor Recalibration Plan would be communicated *‘from time to time’* [65], causing uncertainty about the program. Subsequently, in November 2021, the Home Minister announced that employers in the construction, manufacturing, plantation, and agriculture sectors could legally hire undocumented migrant workers under the Labor Recalibration Plan [66]. But, the Director-General of Immigration later clarified that this program was solely for undocumented workers held in immigration detention centers and that employers intending to hire them would be required to finance the repatriation of these detainees at a one-to-one ratio [67]. However, the Human Resources Minister specified that the policy was intended for foreign workers with valid documents who had lost their jobs due to their employers’ pandemic-related business closures, with no plans to legalize workers who had been undocumented prior to the pandemic [68]. Despite the government’s initial intent to manage these programs directly, recruiting agents and intermediaries soon began offering their services to migrant workers for fees reaching up to RM3,000 (approximately USD 631) [69]. Ultimately, the government acknowledged the program’s lack of success [70]. The government attributed this failure to the age limit requirement for workers to be below 45 years old [70]. However, migrant activists argued that the high cost of hiring a worker (approximately RM 4,000 or USD 952) during an economic downturn, coupled with mixed messages about the arrest and detention of undocumented workers, deterred employers and workers from participating in the program [71].

This lack of policy coherence across various government initiatives left migrants vulnerable, subjected them to heightened security measures by the State, and exposed them to increased xenophobic and racist hate speech on social media. Furthermore, it created uncertainty for employers grappling with severe labor shortages and economic losses. Surveys by the National Chamber of Commerce and Industry of Malaysia (NCCIM) and other industry estimates revealed worker shortages in various sectors during the fourth quarter of 2021, including plantation (70,000), rubber glove manufacturing (25,000), furniture (30,000), construction (200,000), services (45,000), plastics (6,293) [72], cleaning services (68,000), and restaurants (30,000) [73]. While estimates varied among surveys, they all pointed to labor shortages, highlighting the adverse economic consequences of ad-hoc and fragmented policies for migrant workers. This predicament was exacerbated by the fact that small and medium enterprises (SMEs) constituted the majority of businesses in Malaysia, accounting for 98.5% of firms nationally [74] and contributing 38.3% to the country’s GDP and 48% of total employment in 2020 [75]. Markedly, the president of the SME Association of Malaysia noted that only 15% of migrant workers employed in this sector were documented, with the majority being undocumented, often hired without formal employment contracts or receiving only daily wages [76], highlighting the significant problems with the role of migration in the country’s development strategies.

Additionally, the lack of policy coherence hindered the Ministry of Health’s ability to effectively implement an evidence-based public health strategy aimed at combating the pandemic through comprehensive measures such as universal testing, treatment, and vaccination for COVID-19.

The above complex and perplexing situation reveals the presence of competing and conflicting interests and spheres of influence among the State, employers, and intermediaries in the migration industry, all of which play a significant role in shaping the risk and vulnerability of migrant workers to COVID-19.

### Normalization of cheap and disposable migrant labor in neoliberal economic regimes during COVID-19

The salience of cheap and disposable migrant labor in neoliberal economic regimes which existed before COVID-19, became even more pronounced during the humanitarian crisis. As the economy faced challenges, migrant workers were among the first to be laid off [77]. Within a month of lockdowns, migrants were struggling to access food [29]. However, they were excluded from social protection and state-initiated financial assistance programs provided to citizens during the pandemic [5] without regard for their significant economic contributions to the economy [78] or their dire need during a humanitarian emergency.

Common complaints reported by migrant workers during the pandemic included unfair termination, unpaid wages, continuing nonessential work, and uncertainty about their employment status due to limited contact with employers, as noted by the International Labor Organization (ILO) and Malaysian Trades Union Congress (MTUC) [79]. Domestic workers faced additional challenges as work and education moved online, resulting in increased responsibilities, longer hours, uncompensated overtime, limited time off, and difficulties sending remittances back home [79].

Moreover, although the Workers’ Minimum Standards of Housing and Amenities (Amendment) Act 2019 (Act 446) initiated by the government aimed to improve access to safe housing, its enforcement was delayed, and migrant worker accommodations lacked robust monitoring during the pandemic. This resulted in over 90% of foreign workers in Malaysia residing in housing that did not comply with regulations [80]. Malaysian glove manufacturers who supplied 60% of the world’s gloves during the pandemic [81] and the construction sector faced scrutiny for their workers’ substandard living conditions. Top Glove, for example, became Malaysia’s largest COVID-19 cluster, with the majority of cases among its migrant workers. Workers reported overcrowded accommodations, limited sanitation facilities, and poor ventilation [82, 83]. Similar conditions were found on construction sites, with workers living in cramped containers under unsanitary conditions [84]. The national human rights institution of Malaysia, SUHAKAM, in monitoring the needs of vulnerable communities during COVID-19 reported that most migrant workers lived in overcrowded *kongsi* houses (makeshift housing for construction workers) with 40–80 other occupants [27].

Workplace safety was also inadequately regulated, leading to preventable “superspreader zones” [28]. Despite Ministry of Health advisories to companies regarding Standard Operating Procedures (SOP) and preventive measures in the workplace [85], media reports exposed many companies that failed to implement recommended COVID-19 protocols such as sanitization, physical distancing, and mask-wearing [86]. Top Glove, for instance, faced criticism when a migrant worker revealed subpar safety measures and inconsistent enforcement of SOPs. Workers often felt compelled to continue working in these conditions due to debts owed to recruitment agents for their migration to Malaysia. Top Glove terminated the employment of the worker who raised concerns, further highlighting the challenges faced by these workers [82]. Investigations revealed the prevalence of forced labor and harsh working conditions in prominent factories during the pandemic [87, 88]. This issue gained international attention when the U.S. Customs and Border Protection (UCBP) banned the import of disposable rubber gloves from major companies like Top Glove, citing indicators of forced labor [88].

The work conditions in the glove-making industry before the pandemic were already a cause for concern. Investigative reports dating as far back to 2018 highlighted the prevalence of forced labor and deplorable working conditions within this sector. However, in July 2020, following an unscheduled visit, the Ministry of Human Resources cleared one of the companies implicated in the forced labor issue by the U.S. Customs and Border Protection (UCBP). Interestingly, despite the ministry’s clearance, the company chose to compensate its workers who met certain criteria related to recruitment fees. This compensation amounted to a cumulative total of USD 40 million and was aimed at resolving the UCBP ban [89]. This sequence of events suggests that the poor work conditions and inadequate enforcement of workplace safety policies that existed in the glove-making industry before the COVID-19 pandemic played a significant role in exacerbating the health risks faced by migrant workers during the pandemic.

While countries such as Canada, Turkey, and Denmark proactively disseminated COVID-related information in the languages spoken by migrants [90], Malaysia did not provide health information about COVID-19 in the languages understood by its migrant population.

From the examples provided above it emerges that the exceptional circumstances of the COVID-19 crisis entrenched the normalization of the pre-existing neglect of migrant rights and protection, often rooted in their exceptional status as non-citizens.

### Securitization of migration in the context of public health policies

The securitization of migration was a pre-existing phenomenon in Malaysia, but the advent of the pandemic provided an opportunity to further reinforce this securitization, especially in the context of health and COVID-19. Presented as an unprecedented global health crisis and aligning with the WHO's concept of public health security [91], COVID-19 justified the necessity for states to undertake proactive and reactive measures in line with the norm of health security.

The diverse operational interpretations of security, however, exhibited inconsistencies across ministries, as exemplified in this case. In March 2020, despite the then Defense Minister and later Prime Minister, Datuk Seri Ismail Sabri Yaakob, initially reassuring undocumented migrants that they need not fear arrest due to the government’s primary focus on pandemic response [92], this position was reversed within less than a month. Three buildings housing migrant workers in central Kuala Lumpur were placed under total lockdown or an enhanced movement control order (EMCO) as part of targeted cluster identification to stem disease transmission [58], and mass raids and arrests began. Moreover, to prevent the virus from entering the country, various enforcement agencies, including the police, army, Malaysian Maritime Enforcement Agency, Immigration Department, and the People’s Volunteers Corps (RELA), tightened land, sea, and air borders to prevent the possibility of undocumented migrants entering through illegal routes, known as *‘laluan tikus’* or rat lanes [93, 94]. Despite calls from rights groups and public health experts to cease the arrests, Datuk Seri Ismail Sabri Yaakob stated, ‘We arrest them because it’s against the law’ [61]. On October 26, 2020, the Home Ministry revealed that 756 children were being held at immigration detention centers nationwide, of which 405 were held without their parents or guardians [95]. As arrests continued in 2020, the National Human Rights Commission, Suruhanjaya Hak Asasi Manusia Malaysia’s (SUHAKAM), estimated that immigration detention centers, which could only accommodate 12,530 detainees, had exceeded their capacity by 20 percent, with as many as 15,163 detainees being held [96].

The contradictory statements and policy reversals regarding the arrest and detention of undocumented migrants during the peak of the pandemic gave rise to various public health issues. First, following mass arrests in April and May 2020, new clusters of coronavirus infections were detected at three immigration detention centers [97], with similar clusters continuing to emerge in other detention centers. The Bukit Jalil Immigration Detention Center cluster was the first to be detected primarily comprising migrant detainees who had come into contact with a COVID-19-positive case In one detention center, almost 40 percent of the detainees tested positive for COVID-19 [98]. The Ministry of Health identified immigration detention centers as ‘high-risk areas’ [38, 99], and targeted screening and sampling of detainees and staff commenced, as the rapid transmission of the disease was attributed to the overcrowded and confined conditions within these centers [100, 101]. These arrests also contributed to vaccine hesitancy among undocumented migrants [62] This hesitancy emerged as the Ministry of Health aimed to maximize vaccination coverage as a strategy to attain herd immunity, a crucial step in transitioning the nation toward endemicity.

COVID-19 also exposed and exacerbated overt xenophobia, racial discrimination, and ethnic biases in Malaysia undermining the effort to fight the pandemic in the country. Notably, the arrival of a boat carrying Rohingya asylum seekers in Malaysian waters in mid-April 2020, subsequently being turned away and escorted by two Navy vessels after providing sustenance to those on board [102], with the emergence of COVID-19 cases within detention centers, triggered a surge in hate speech targeting migrants, refugees [103] and human rights advocates advocating for their welfare [104]. Remarkably, despite this backdrop, the government remained conspicuously silent on the issue of hate speech and violent threats directed at migrants and refugees [105]. Furthermore, Reuters reported the existence of over 36 pages and groups, some linked to current and former Malaysian security officials, which contained discriminatory content directed towards Rohingya refugees and undocumented migrants [106]. In response to this concerning development on hate speech against migrants, the Director-General of Health, cautioned against healthcare discrimination targeting migrants [107].

### Lessons learned

Based on the identified key issues related to policies concerning migrant workers during the COVID-19 pandemic in Malaysia, two important lessons emerge:

1. The necessity for a paradigm shift in migration governance discourse.
2. The importance of the Health in all Policies (HiAP) approach.

#### The necessity for a paradigm shift in the migration governance discourse

This imperative arises from the pre-existing disparities in the social determinants of health of migrants before the onset of the COVID-19 pandemic, leading to unequal exposure to SARS-CoV-2. The problem originates from unquestioned neoliberal norms that underlie both migration and development. These norms idealize a ‘good migrant,’ regardless of their status or conditions, as someone who is ‘law-abiding, adaptable to market demands, and eager to contribute to the development of their country of origin’ [108] (p.434). Furthermore, the neoliberal norms of autonomy and individual responsibility that underlie both migration and development normalize the compelling of migrants to maintain their status and functioning even in situations characterized by weak labor and social protection policies or lax enforcement of policies and laws. This compels migrants to persevere under adverse conditions, often without adequate safeguards or recourse.

A recent International Organization for Migration (IOM) published study [109] that investigates the process of migrants becoming undocumented in Malaysia sheds light on the pivotal role played by exploitative employment conditions. These conditions encompass various issues, including inadequate wages and compensation, substandard living conditions, the absence of health and safety regulations and practices, both prior to and during the pandemic, and excessively long working hours. Additionally, deceptive practices carried out by recruitment agencies and employers further compound the problem. When migrants opt to leave such exploitative working conditions, they inadvertently transition into undocumented status because their residency and work permits are intricately tied to their employers under existing laws. Viewed from this perspective, the problem of undocumented or irregular migration is not primarily a security issue, but an administrative issue that can be solved with appropriate policies and mechanisms, although there are security dimensions to irregular migration. Effectively addressing these systemic disparities necessitates a fundamental shift towards the principles of structural justice in migration governance.

In light of the circumstances, it is imperative to reconsider the tagline of “safe, orderly, regular migration,” which was initially promoted by the International Organization for Migration (IOM), used in discourses on the Sustainable Development Goals (SDGs), and more recently adopted by the World Health Organization (WHO) as a comprehensive and inclusive response to COVID-19 [90] (p.34). Instead, the appropriate policy approach should be ‘safe, just, and regular migration,’ as the absence of justice and respect for human rights jeopardizes human security and health everywhere. Without an internationally agreed-upon definition of safe and orderly migration [110], state-centric discourses surrounding these terms presume and reinforce subjectivities that prioritize regular migration as a prerequisite for safety and well-being in the context of migration. While regular migration is undoubtedly important, assuming safety in regular migration, viewed as orderly, can be disputed due to the fluidity of migration statuses, such as ‘regular’/‘irregular’ or ‘documented’/‘undocumented,’ during the migration process. Regular and orderly migration does not inherently guarantee the safety or health of migrants, particularly considering the framing and implementation of current migrant policies that create precariousness and risks, even for documented migrants, as this case study has demonstrated. When state policies disregard human rights, eschew inclusive approaches, and perpetuate inequalities, both the safety and health of migrant populations are compromised, even if their status is regular. COVID-19 has accentuated this reality most poignantly.

Thus, the establishment of sustainable pathways for migrants to pursue safe, just, and regular migration is crucial for safeguarding the health and well-being of both migrants and host populations. In the Malaysian context, this entails the development of a comprehensive and coherent migration policy that harmonizes and integrates diverse forms of mobility and administrative mechanisms tailored to different migrant categories. This approach also necessitates the reinforcement of workplace safety regulations and the protection of workers’ rights, including mechanisms for addressing wage theft and unjust termination. Additionally, it involves rigorous monitoring and oversight of migrant workers’ housing conditions, as well as ensuring access to healthcare and social protection, both in ordinary circumstances and during pandemics.

In addition to the aforementioned considerations, the implementation of more suitable migrant health policies can also mitigate the transmission of the pathogen to the general population and hold a broader societal impact, extending beyond migrant communities. The beneficial outcomes include the potential to reduce disease transmission, expedite herd immunity, prevent future disease resurgences, bolster economic productivity, enhance public health system resilience, and promote social cohesion, underscoring the importance of equitable and comprehensive migrant policies in safeguarding the health and well-being of both migrants and the host populations.

#### The importance of the Health in all Policies (HiAP) approach

The divergent approaches to migrant policies in relation to COVID-19 are rooted in contrasting perspectives that juxtapose universal approaches to testing, treatment, and care with the securitization of migration and health. As an unparalleled global health emergency, COVID-19 reinforced the need for states to undertake extraordinary measures in line with the norm of health security, to ‘minimize vulnerability to acute public health events that endanger the collective health of populations living across geographical regions and international boundaries’ [91] (p.ix).

Explaining the mechanism of norm diffusion through the case of health securitization from a global to local context, Kaunert, Leonard, and Wertman [111] provide a valuable framework for understanding how global norms can undergo modifications in content and normative foundations when implemented locally. They argue that the adaptation of global norms at the local level is influenced by pre-existing domestic norms, which serve as interpretative lenses for the core global norm. This adaptation is also shaped by the contextual factors from which the norm originates and the agent and strategies of the norm’s promoter. They further emphasize that primary norms encounter profoundly contested normative terrains when applied at the local level. In the context of COVID-19, where the securitization of health encompasses the primary norm of disease containment to mitigate harm and vulnerability among populations, the initiation of this norm was led by international entities such as the World Health Organization (WHO). However, the specific manifestations of this primary norm in addressing COVID-19 for migrant populations varied across different countries, contingent upon local circumstances and the actors responsible for translating these norms. For example, across 53 jurisdictions, governments aimed to mitigate overcrowding and minimize COVID-19 transmission in incarcerated settings by reducing prison populations via the release of both prisoners and pre-trial detainees, restricting new admissions into correctional systems, and implementing temporary releases, where prisoners were allowed to leave confinement for a specified duration, with an obligation to return to prison afterward [112]. Similarly, other good practice policies included considering undocumented migrants pending regularization as regular migrants (Peru), providing limited amnesty (Kuwait, Bahrain), waiving of sanctions and financial penalties (South Africa), and suspension of forced return (Canada, Chile, Czechia, Finland, Ireland, Latvia, Lithuania, Luxembourg, Malta, the Russian Federation, Slovakia, and Spain) [90]. On the other hand, in Malaysia, despite the international community discouraging a punitive approach toward migrant populations [113], the alignment of health securitization and the response to COVID-19 with the securitization of migration yielded a series of stringent measures including the mass apprehension and incarceration of migrant workers, and the repatriation of undocumented migrants to reduce COVID-19 transmission. Scholars note that discourses framing migration as a security concern are frequently accompanied by acts of violence, threats, and xenophobic rhetoric directed at migrant communities [114], the phenomenon of hate speech being observed in Malaysia.

The above critique of the securitization of migration and migrant health during COVID-19 does not undermine the importance of the state’s role in safeguarding and upholding the territorial integrity and sovereignty of the nation or in addressing irregular or undocumented migration, as these are legitimate responsibilities of the state. However, it prompts the question of the extent to which undocumented or irregular migration should be perceived as a security issue as opposed to an administrative one? Could a change in framing lead to a resolution of a longstanding issue? Would the development of a coherent migration policy, accompanied by effective administrative mechanisms targeting the underlying causes of documented workers transitioning to undocumented status, offer a solution to the problem of undocumented migrants in the country? Given the pivotal role that migration has played in the country’s development for decades, it raises concerns that only 15% of migrant workers employed in the SME sector, which comprises over 95% of the country’s businesses, contributes to more than a third of the GDP and nearly half of the total employment, possess proper documentation. This issue becomes particularly significant when considering that approximately 17 ministries are involved in the SME sector development [115]. While these questions prompt contemplation on how irregular migration after arrival in the country can be curtailed, with regards to border enforcement, could a proactive approach to securing the borders, rather than accepting porous borders as an inevitability, help in stemming irregular entry into the country? Should migrants be held responsible for porous borders? These inquiries hold significance due to the fragile ethical basis upon which the argument for the securitization of migration rests, especially when all the evidence and arguments pertaining to the development of undocumented migration and the ramifications of securitization of migration on their human security is taken into account.

Returning to the discussion on COVID-19 and migrants in Malaysia, the emergence of clusters in detention centers, coupled with the disregard for workplace safety, overcrowded living conditions, and inadequate sanitation and water supply, underscores the profound impact that policies in non-health sectors can exert on health outcomes through intricate and multifaceted pathways. This underscores the critical significance of the Health in All Policies (HiAP) approach. In fact, closer home, the Singaporean context serves to reinforce this idea. Migrant laborers residing in dormitories at the lower end of the wage spectrum in Singapore experienced a disproportionate impact from the COVID-2019. This disparity was linked to their communal living in high density and unsanitary dormitory facilities, as well as the absence of comprehensive protection mechanisms [116].

HiAP is ‘an approach to public policies across sectors that systematically takes into account considers the health implications of decisions, seeks synergies, and avoids harmful health impacts to improve population health and health equity’ [117]. Via a whole-of-government approach, HiAP seeks to bring about a collaborative and coordinated response, particularly crucial for facilitating intersectoral coordination during crises. Equally, the migrant experience of the pandemic also emphasizes the critical importance of a whole-of-society approach to pandemic preparedness [118] so that greater efficiency and effectiveness can be achieved through the coordinated response of governments, businesses, and civil society. Such an approach is guided by robust governmental leadership and complemented by the involvement of civil society organizations that possess the capacity to transform scientific and government data into actionable strategies, largely due to the trust they have established with various constituencies. Employers also play a pivotal role in promoting workplace safety, disseminating accurate and timely information, and safeguarding employee health. However, in the case of migrant populations, the requisite inclusivity in a whole-of-society approach is frequently compromised because migrants, as non-citizens, are often perceived as ‘the other’ and subjected to moral judgments regarding their entitlement to healthcare and other protections, which are otherwise considered universal entitlements for citizens [119]. This case study reinforces the global lesson that equity and inclusivity are fundamental components of effective pandemic control [120] and the journey toward achieving equity must commence before a pandemic emerges. Only through the establishment of such structural prerequisites can migrants experience reduced vulnerability to pathogen exposure, gain access to health information and services, and enjoy occupational health safeguards and worker protection.

## Conclusion

This paper sought to examine how migrants were considered in the COVID-19 policies of the Malaysian government and the lessons learned that could inform future pandemic preparedness. The findings from the review unravel complex inter-linkages between the contexts of migration and health which coalesce to engender and exacerbate vulnerability to disease and ill-health for the migrant workers in times of pandemic. Gaps in coordination and incoherence in policies addressing migrant workers during the pandemic, the normalization of cheap and disposable labor in neoliberal economic regimes, and the securitization of migration were key factors contributing to the failure of migration policies to provide protection to migrant workers during COVID-19. The review suggests that policy approaches embodying the principles of Health in All Policies (HiAP), a whole-of-society approach and the promotion of safe, just, and regular migration, predicated on equity and inclusion, are integral to a comprehensive and effective response to pandemics such as COVID-19. To ensure an effective response to pandemics and enhance preparedness for future outbreaks, these concerns must be acknowledged and addressed prior to the emergence of another pandemic.

## Data Availability

Not applicable (The findings of this study are supported by secondary data which are included within the article and/or in the public domain as per the references and links provided).

## Abbreviations

BID: Brought in Dead
EMCO: Enhanced Movement Controlled Orders
GDP: Gross Domestic Product
HiAP: Health in all Policies
ICU: Intensive Care Unit
ILO: International Labor Organization
IOM: International Organization for Migration
MAEPS: Malaysia Agro Exposition Park Serdang
MTUC: Malaysian Trades Union Congress
MCO: Movement Control Orders
NCCIM: National Chamber of Commerce and Industry of Malaysia
POC: Persons of Concern
PPE: Personal Protective Equipment
PSWS: Persatuan Sahabat Wanita Selangor
SMEs: Small And Medium Enterprises
SOCSO: Social Security Organisation
SUHAKAM: Malaysian National Human Rights Organization (Suruhanjaya Hak Asasi Manusia Malaysia)
SDGs: Sustainable Development Goals
TIP: Trafficking in Persons
TPVA: Trafficking Victims Protection Act’s
UCBP: U.S. Customs and Border Protection
UNHCR: United Nations High Commissioner for Refugees
WHO: World Health Organization

## References

[1] Bojorquez I, Cabieses B, Arósquipa C, Arroyo J, Novella AC, Knipper M (2021). Migration and health in Latin America during the COVID-19 pandemic and beyond. The Lancet.

[2] Tazreiter C, Metcalfe S (2021). New vulnerabilities for migrants and refugees in state responses to the global pandemic, COVID-19. Soc Sci.

[3] Wahab A (2020). The outbreak of Covid-19 in Malaysia: pushing migrant workers at the margin. Soc Sci Humanit Open.

[4] Anam MZ, Warsito T, Al-Fadhat F, Pribadi U (2021). COVID-19 and decent work: online media coverage on Indonesian female migrant domestic workers in Malaysia and Taiwan. Sociol Tecnociencia.

[5] Aziz SNA, Basir SM, Mahalingam M (2022). Job termination and social security of migrant workers in Malaysia during Covid-19 pandemic. Padjadjaran Jurnal Ilmu Hukum.

[6] Khoo LS, Hasmi AH, Ibrahim MA, Mahmood MS (2020). Management of the dead during COVID-19 outbreak in Malaysia. Forensic Sci Med Pathol.

[7] Ng SH (2022). Health inequalities amongst refugees and migrant workers in the midst of the COVID-19 pandemic: a report of two cases. Asian Bioeth Rev.

[8] Ngan OMY, Sanip S (2021). The vulnerability of migrant workers in global COVID-19 pandemic: highlights from Malaysia and Hong Kong. Asia Pac J Public Health.

[9] Rashid AA, Mohamad I, Haris AFM (2021). The role of social media in making an impact to health knowledge and behaviour on covid-19 in Malaysia. Malays J Med Sci.

[10] Saidin MIS, Omar HB (2021). COVID-19 policy response and the vulnerabilities of low-skilled women migrant workers in Malaysia. Round Table.

[11] Pillai P (1999). The Malaysian State’s response to migration. Sojourn.

[12] International Organization for Migration. Internatonal dialogue on migration. Challenges of irregular migration: addressing mixed migration flows, MC/INF/294. Discussion note, para 6. United Nations; 2008. Cited 2023 Oct 20.

[13] International Organization for Migration. International migration law N°25 - glossary on migration. 2nd ed. Geneva: IOM; 2011. Available from: https://publications.iom.int/system/files/pdf/iml25_1.pdf. Cited 2023 Oct 20.

[14] MOHR. Labor migration trends, policy and regulatory changes: Malaysia (2019–2022). Ministry of Human Resources Malaysia; 2023.

[15] World Bank. Who is keeping score? Estimating the number of foreign workers in Malaysia. The Malaysian development experience Malaysia. World Bank; 2020. Available from: https://openknowledge.worldbank.org/handle/10986/33730. License: CC BY 3.0 IGO. Cited 03 dec 20201.

[16] UNDESA. International migrant stock: By destination and origin New York, NY. UN, Population Division; 2019. Available from: https://population.un.org/wpp/Download/Standard/Population. Cited 2021 Apr 20.

[17] Lee HA, Khor Y (2018). Counting migrant workers in Malaysia: a needlessly persisting conundrum. Issue 25. Contract No.: 25.

[18] UNHCR. Figures at a glance in Malaysia. Kuala Lumpur; 2022. Available from: https://www.unhcr.org/en-my/figures-at-a-glance-in-malaysia.html. Cited 2022 June 09.

[19] Todd L, Amirullah A, Shin WY. The economic impact of granting refugees in Malaysia the right to work. Institute for Democracy and Economic Affairs; 2019.

[20] Task Force on Measuring Circular Migration. Defining and measuring circular migration ECE/CES/STAT/2016/5 New York and Geneva: United Nations; 2016. Available from: https://unece.org/fileadmin/DAM/stats/publications/2016/ECECESSTAT20165_E.pdf. Cited 2023 Oct 19.

[21] Kaur A (2004). Crossing frontiers: race, migration and borders in Southeast Asia. Int J Multi Soc.

[22] Kuala Lumpur Hospital. Hospital charges. Kementerian Kesihatan Malaysia; 2023. Available from: https://hkl.moh.gov.my/index.php/advanced-stuff/hospital-charges. Cited 27 July 2023.

[23] Loganathan T, Rui D, Ng CW, Pocock NS (2019). Breaking down the barriers: Understanding migrant workers’ access to healthcare in Malaysia. PLoS ONE.

[24] MOH. Pekeliling ketua pengarah kesihatan bil. 10/2001. Garispanduan melaporkan pendatang tanpa izin yang mendapatkan perkhidmatan kesihatan di hospital dan klinik kesihatan. Ruj Kami: Bil (1)dlm.KKM/62/BPKK(AM)/Pel-22. Pejabat Ketua Pengarah Kesihatan Malaysia; 2001.

[25] United Nations. CEDAW/C/MYS/CO/3–5: concluding observations on the combined third to fifth periodic reports of Malaysia. Office of the High Commissioner for Human Rights; 2018. Available from: https://www.ohchr.org/en/documents/concluding-observations/cedawcmysco3-5-concluding-observations-combined-third-fifth. Cited 2023 June 22.

[26] The Star Online. Human Resources Ministry releases FAQ during MCO period. 2020. Available from: https://www.thestar.com.my/news/nation/2020/03/20/human-resources-ministry-releases-faq-during-mco-period#.XnVmWDiAIcw.whatsapp. Cited 12 Dec 2020.

[27] SUHAKAM. Dialogue with vulnerable communities: an assessment of needs and next steps amid COVID-19 pandemic. SUHAKAM; 2020. Available from: https://suhakam.org.my/dialogue-with-vulnerable-communities-an-assessment-of-needs-and-next-steps-amid-COVID-19-pandemic/. Cited 11 Dec 2021.

[28] Wahab A (2020). Migrant workers and Covid-19 outbreak in Malaysia.

[29] Priya SS. Migrant workers and refugees cry for help. 2020. Available from: https://www.thestar.com.my/metro/metro-news/2020/04/09/migrant-workers-and-refugees-cry-for-help. Cited 11 Dec 2021.

[30] Xavier I. Comment | Giving birth in a kongsi during lockdown. Malaysiakini; 2020. Available from: https://www.malaysiakini.com/news/521606. Cited 12 Dec 2020.

[31] FMT. 3 govts agree to take back Covid-free migrants. FMT; 2020. Available from: https://www.freemalaysiatoday.com/category/nation/2020/05/28/3-govts-agree-to-deportation-of-covid-free-illegals/. Cited 12 Dec 2021.

[32] Sukumaran T, Jaipragas B. Coronavirus: hundreds arrested as Malaysia cracks down on migrants in Covid-19 red zones. South China Morning Post. Available from: https://www.scmp.com/week-asia/politics/article/3082529/coronavirus-hundreds-arrested-malaysia-cracks-down-migrants. Cited 02 Dec 2021.

[33] Daud S (2021). The COVID-19 pandemic crisis in Malaysia and the social protection program. J Dev Soc.

[34] Verghis S, Pereira X, Kumar A, Koh A, Singh-Lim A (2021). COVID-19 and refugees in Malaysia: an NGO response. Intervention.

[35] Khalidi JR, Noor NM. COVID-19 and the myth of the ‘dirty foreigner’ in Malaysia. Centre for Southeast Studies, Kyoto University; 2020. Available from: https://covid-19chronicles.cseas.kyoto-u.ac.jp/en/post-061-html/. Cited 25 Dec 2021.

[36] MOH. COVIDNOW. COVID-19 Cases in Malaysia. Last updated: 10 Dec 2021. Ministry of Health, Malaysia; 2021. Available from: https://covidnow.moh.gov.my/cases/. Cited 10 December 2021.

[37] MOH. Official Github account of Malaysia’s Ministry of Health. Presently focused on COVID-19 data, in collaboration with the COVID-19 Immunisation Task Force. Ministry of Health; 2021. Available from: https://github.com/MoH-Malaysia/covid19-public/blob/main/epidemic/clusters.csv. Cited 10 Dec 2021.

[38] Fishbein E, Hkawng J. Immigration detention centres become Malaysia coronavirus hotspot. Aljazeera; 2020. Available from: https://www.aljazeera.com/news/2020/6/2/immigration-detention-centres-become-malaysia-coronavirus-hotspot. Cited 10 Dec 2021.

[39] BBC. Malaysia migrant raids ‘to reduce Covid-19 spread’. BBC; 2020. Available from: https://www.bbc.com/news/world-asia-52515000. Cited 02 Dec 2021.

[40] Buzan B, Waever O, de Wilde J (1998). Security: a new framework for analysis.

[41] Loheswar R. Health experts: Spraying migrants with disinfectant unscientific defence against Covid-19, likely to cause harm instead. Malay Mail; 2020. Available from: https://www.malaymail.com/news/malaysia/2021/06/10/health-experts-spraying-migrants-with-disinfectant-unscientific-defence-aga/1980991. Cited 12 Dec 2021.

[42] World Bank. Malaysia. Estimating the number of foreign workers. (A report from the Labor Market Data for Monetary Policy task). World Bank; 2019. Available from: https://documents1.worldbank.org/curated/en/953091562223517841/pdf/Malaysia-Estimating-the-Number-of-Foreign-Workers-A-Report-from-the-Labor-Market-Data-for-Monetary-Policy-Task.pdf. Cited 02 Dec 2021.

[43] Salim S. Covid-19: Over 1,500 workplace clusters detected in past five months; 133,247 staff tested positive, 370 deaths. The Edge Markers; 2021. Available from: https://www.theedgemarkets.com/article/covid19-over-1500-workplace-clusters-detected-past-five-months-133247-staff-tested-positive. Cited 11 Dec 2021.

[44] Karupiah S (2021). Impact of COVID-19 on migrant workers from the trade union experience.

[45] Arumugam T, Babulal V. Covid-19 situation not improving, say experts. New Straits Times; 2021. Available from: https://www.nst.com.my/news/nation/2021/08/720095/covid-19-situation-not-improving-say-experts. Cited 10 Dec 2021.

[46] MOH. Classification of COVID-19 Death for National COVID-19 Death Statistics Ministry of Health Malaysia. Ministry of Health Malaysia Ministry of Health; 2020. Available from: https://covid-19.moh.gov.my/garis-panduan/garis-panduan-kkm/ANNEX-45-CLASSIFICATION-OF-COVID19-DEATH-14032022.pdf.

[47] Lim PY, Md Said S, Kadir Shahar H, Azman AZF, Mokhtar SA, Mahmud A (2022). COVID-19 inpatient deaths and brought-in-dead cases in Malaysia. Front Public Health.

[48] United Nations. International convention on the protection of the rights of all migrant workers and members of their families. Geneva: United Nations; 1990. Available from: https://www.ohchr.org/en/professionalinterest/pages/cmw.aspx. Cited 20 Jan 2022. 12318128

[49] Adi N, Noor H, Kamaruddin N (2017). Fundamental rights of domestic workers in Malaysia: Special Reference to the Employment Act 1955 and International Labour Organization Convention (ILO) No. 189. Adv Sci Lett.

[50] Arksey H, O’Malley L (2005). Scoping studies: towards a methodological framework. Int J Soc Res Methodol.

[51] Levac D, Colquhoun H, O’Brien K (2010). Scoping studies: advancing the methodology. Implement Sci.

[52] Tricco AC, Lillie E, Zarin W, O’Brien KK, Colquhoun H, Levac D (2018). PRISMA extension for scoping reviews (PRISMA-ScR): Checklist and explanation. Ann Intern Med.

[53] Grimshaw J. A guide to knowledge synthesis. Canadian Institutes of Health Research; 2010. Available from: https://cihr-irsc.gc.ca/e/41382.html. Cited 02 February 2022.

[54] Peters MDJ, Marnie C, Colquhoun H, Garritty CM, Hempel S, Horsley T (2021). Scoping reviews: reinforcing and advancing the methodology and application. Syst Rev.

[55] Braun V, Clarke V (2006). Using thematic analysis in psychology. Qual Res Psychol.

[56] CodeBlue. Health DG says free covid-19 tests, treatment for foreigners, after pm says payments required. Galen Centre; 2020. Available from: https://codeblue.galencentre.org/2020/03/23/health-dg-says-free-covid-19-tests-treatment-for-foreigners-after-pm-says-payments-required/. Cited 11 Dec 2020.

[57] Bedi RS, Anis MN. Covid-19 screening: Socso coverage for foreign workers who contribute to organisation. The Star; 2020. Available from: https://www.thestar.com.my/news/nation/2020/05/05/covid-19-screening-socso-coverage-for-foreign-workers-who-contribute-to-organisation. Cited 12 Dec 2021.

[58] Ravindran S. Covid-19: 79 cases found in the three KL locations under enhanced MCO, says FT Minister. The Star; Shalini Ravindran. Available from: https://www.thestar.com.my/news/nation/2020/04/10/covid-19-79-cases-found-in-the-three-kl-locations-under-enhanced-mco-says-ft-minister. Cited 10 Dec 2021.

[59] Sherman R, David N. Malaysia: Mass arrests of undocumented migrants to ensure they get COVID-19 jabs. Benar News; 2021. Available from: https://www.benarnews.org/english/news/malaysian/my-migrants-06032021162743.html. Cited 10 Dec 2021.

[60] Sukumaran T, Jaipragas B. Coronavirus: hundreds arrested as Malaysia cracks down on migrants in Covid-19 red zones. South China Morning Post; 2020. Available from: https://www.scmp.com/week-asia/politics/article/3082529/coronavirus-hundreds-arrested-malaysia-cracks-down-migrants. Cited 02 Dec 2021.

[61] CodeBlue. Ismail Sabri doubles down on nabbing undocumented immigrants. Galen Centre; 2020. Available from: https://codeblue.galencentre.org/2020/05/26/ismail-sabri-doubles-down-on-nabbing-undocumented-immigrants/. Cited 10 Dec 2021.

[62] Fishbein E, Hkawng JT. Fear of arrest among undocumented risks Malaysia vaccine push. Aljazeera; 2021. Available from: https://www.aljazeera.com/news/2021/8/6/mixed-messaging-in-malaysia-leaves-migrants. Cited 11 Dec 2021.

[63] JIM. Immigration Department of Malaysia. Frequently asked questions (FAQ). Recalibration program (Repatriation). Jabatan Imigresen Malaysia; 2020.

[64] JIM. Jabatan Imigresen Malaysia. Soalan – soalan lazim. Program rekalibrasi tenaga kerja. 17 November 2020. Jabatan Imigresen Malaysia; 2020. Available from: https://www.imi.gov.my/~johor/borang/FAQs_REKALIBRASI_TENAGA_KERJA_V4_24112020.pdf. Cited 21 March 2022.

[65] Anis MN. Immigration DG: employers participating in Labour Recalibration Plan must ensure they are not blacklisted for immigration offences. The Star; 2020. Available from: https://www.thestar.com.my/news/nation/2020/11/13/immigration-dg-employers-participating-in-labour-recalibration-plan-must-ensure-they-are-not-blacklisted-for-immigration-offences. Cited 11 Dec 2021.

[66] InterManpower. Labour Recalibration Plan to legalize undocumented immigrants. 2020. Available from: https://www.intermanpower.com/2020/11/13/labour-recalibration-plan-to-legalize-undocumented-immigrants/. Cited 11 Dec 2020.

[67] Anis MN. Not all eligible for recalibration plan. The Star; 2020. Available from: https://www.thestar.com.my/news/nation/2020/12/05/not-all-eligible-for-recalibration-plan. Cited 11 Dec 2021.

[68] Bernama. Govt introduced recalibration programmes, not legalising foreign workers, says Saravanan. Astro Awani; 2021. Available from: https://www.astroawani.com/berita-malaysia/govt-introduced-recalibration-programmes-not-legalising-foreign-workers-saravanan-333777. Cited 11 Dec 2020.

[69] Paul J. Letter | fine-tune labour recalibration programmes. Malaysiakini; 2020. Available from: https://www.malaysiakini.com/letters/554450. Cited 13 dec 2021.

[70] Bernama. Hamzah: employers must bear costs of bringing in foreign workers. New Straits Times; 2021. Available from: https://www.nst.com.my/news/nation/2021/10/739891/hamzah-employers-must-bear-costs-bringing-foreign-workers. Cited 11 Dec 2021.

[71] Khoo I. Raids make migrant legalisation plan a failure. FMT; 2021. Available from: https://www.freemalaysiatoday.com/category/nation/2021/01/25/raids-make-migrant-legalisation-plan-a-failure/. Cited 11 Dec 2021.

[72] Lai A, Lee S, Devi V, Yun YX. Extend deadline for recalibration plan, say business groups. The Star; 2021. Available from: https://www.thestar.com.my/news/nation/2021/10/27/extend-deadline-for-recalibration-plan-say-business-groups. Cited 11 Dec 2021.

[73] Khoo I. Lift ban on foreign workers and simplify recalibration, govt told. FMT; 2021. Available from: https://www.freemalaysiatoday.com/category/nation/2021/09/30/lift-ban-on-foreign-workers-and-simplify-recalibration-govt-told/. Cited 11 Dec 2021.

[74] SMECORP. Economic Census 2016: Profile of SMEs. 2017. Available from: https://www.smecorp.gov.my/images/SMEAR/latest/2/Census%20English_FINAL.pdf.

[75] DOSM. Small and Medium Enterprises (SMEs) performance 2020. Department of Statistics Malaysia; 2021. Available from: https://www.dosm.gov.my/v1/index.php?r=column/cthemeByCat&cat=159&bul_id=KzdrS25pRTZ1VGFkcTlNY0FEczBYUT09&menu_id=TE5CRUZCblh4ZTZMODZIbmk2aWRRQT09. Cited 11 Dec 2021.

[76] Malaysiakini. ‘Majority of migrant workers hired by SMEs on daily wage, no contract’. 2020. Available from: https://www.malaysiakini.com/news/519299. Cited 11 Dec 2020.

[77] The Star. Human Resources Ministry releases FAQ during MCO period. The Star; 2020. Available from: https://www.thestar.com.my/news/nation/2020/03/20/human-resources-ministry-releases-faq-during-mco-period#.XnVmWDiAIcw.whatsapp. Cited 12 Dec 2021.

[78] Moreno RM, et al. Malaysia economic monitor. Immigrant labor. World Bank; 2015.

[79] ILO (2020). Protecting the rights of domestic workers in Malaysia during the COVID-19 pandemic and beyond.

[80] Thomas J. Govt accused of bowing to businesses with U-turn on workers’ housing. FMT; 2020. Available from: https://www.freemalaysiatoday.com/category/nation/2021/04/23/govt-accused-of-bowing-to-businesses-with-u-turn-on-workers-housing/. Cited 11 December 2021.

[81] Zaugg J. The world’s top suppliers of disposable gloves are thriving because of the pandemic. Their workers aren’t. CNN Business; 2020. Available from: https://edition.cnn.com/2020/09/11/business/malaysia-top-glove-forced-labor-dst-intl-hnk/index.html. Cited 2021 Dec 13.

[82] Lee L, Ananthalakshmi A. Malaysia’s Top Glove fired whistleblower before virus outbreak. Reuters; 2020. Available from: https://www.reuters.com/article/us-health-coronavirus-top-glove-insight-idUSKBN28N005. Cited 11 Dec 2021.

[83] Peter Z. Malaysia’s COVID woes spotlight ‘terrible’ migrant worker housing. Voice of America; 2020. Available from: https://www.voanews.com/a/east-asia-pacific_malaysias-covid-woes-spotlight-terrible-migrant-worker-housing/6199239.html. Cited 11 Dec 2021.

[84] Krishnan DB. Foreign workers’ cramped, rundown kongsi linked to Covid surge. New Straits Times; 2020. Available from: https://www.nst.com.my/news/nation/2020/11/641706/foreign-workers-cramped-rundown-kongsi-linked-covid-surge. Cited 11 Dec 2021.

[85] MOH. Updates on the coronavirus disease 2019 (COVID-19). Situation in Malaysia. Ministry of Health; 2020. Cited 11 Dec 2020.

[86] Szeto E, Taylor C, Tomlinson A. Hidden camera reveals ‘appalling’ conditions in overseas PPE factory supplying Canadian hospitals, expert says. CBC News; 2021. Available from: https://www.cbc.ca/news/world/marketplace-overseas-personal-protective-equipment-manufacturing-working-conditions-1.5873213. Cited 11 Dec 2021.

[87] Ananthalakshmi A, Lee L. Dyson splits with Malaysia supplier, stoking concern over migrant worker treatment. Reuters; 2021. Available from: https://www.reuters.com/business/dyson-splits-with-malaysia-supplier-stoking-concern-over-migrant-worker-2021-12-05/. Cited 11 Dec 2021.

[88] UCBP. CBP issues forced labor finding on Top Glove Corporation Bhd. March 29 2021. U.S. Customs and Border Protection; 2021. Available from: https://www.cbp.gov/newsroom/national-media-release/cbp-issues-forced-labor-finding-top-glove-corporation-bhd. Cited 11 Dec 2021.

[89] Pong A. What Malaysia’s biggest COVID-19 cluster reveals about migrants’ working conditions. The Organization for World Peace; 2020. Available from: https://theowp.org/reports/what-malaysias-biggest-covid-19-cluster-reveals-about-migrants-working-conditions/. Cited 12 Dec 2021.

[90] WHO (2021). Refugees and migrants in times of COVID-19: mapping trends of public health and migration policies and practices.

[91] WHO. The World Health Report 2007 - The world health report 2007 : a safer future : global public health security in the 21st century. World Health Organization; 2007. Available from: https://apps.who.int/iris/bitstream/handle/10665/43713/9789241563444_eng.pdf?sequence=1&isAllowed=y. Cited 22 July 2023.

[92] The Star. Covid-19: illegal migrants at tabligh event can test without fear. 2021. Available from: https://www.thestar.com.my/news/nation/2020/03/22/covid-19-illegal-migrants-at-tabligh-event-can-test-without-fear. Cited 10 Dec 2021.

[93] Ibrahim MI, Parzi MN. COVID-19: Warga asing guna ‘laluan tikus’ langgar PKP. Berita Harian; 2020. Available from: https://www.bharian.com.my/berita/nasional/2020/03/670339/covid-19-warga-asing-guna-laluan-tikus-langgar-pkp. Cited 02 Dec 2021.

[94] Shakirah M, Ang Z, Anis-Syakira J, Cheah K, Kong Y, Selvarajah S (2020). The COVID-19 chronicles of Malaysia | In the face of a pandemic.

[95] Tan T, Sivanandam H, Rahim R. Home ministry: 756 children held at immigration detention centres nationwide as of Oct 26. The Star; 2021. Available from: https://www.thestar.com.my/news/nation/2020/11/04/home-ministry-756-children-held-at-immigration-detention-centres-nationwide-as-of-oct-26. Cited 11 Dec 2021.

[96] The Star. Suhakam: Address crowded prisons. 2020. Available from: https://www.thestar.com.my/news/nation/2020/11/13/suhakam-address-crowded-prisons. Cited 10 Dec 2021.

[97] Kanyakumari D. COVID-19: New cluster involving immigration detainees identified in Malaysia Channel News Asia; 2021. Available from: https://www.straitstimes.com/asia/se-asia/coronavirus-breaks-out-at-detention-centres-in-malaysia-health-ministry-warns-against. Cited 10 Dec 2021.

[98] CodeBlue. MOH reports 270 Covid-19 cases at immigration detention depot. Galen Centre; 2020. Available from: https://codeblue.galencentre.org/2020/06/04/moh-reports-270-covid-19-cases-at-immigration-detention-depot/. Cited 10 Dec 2021.

[99] Fishbein E, Hkawng JT. Immigration detention centres become Malaysia coronavirus hotspot. Aljazeera; 2020. Available from: https://www.aljazeera.com/news/2020/6/2/immigration-detention-centres-become-malaysia-coronavirus-hotspot. Cited 10 Dec 2021.

[100] Ang ZY, Cheah KY, Shakirah MS, Fun WH, Anis-Syakira J, Kong Y-L (2021). Malaysia’s health systems response to COVID-19. Int J Environ Res Public Health.

[101] Soo WJ. Dr Noor Hisham: Confined spaces likely cause of Covid-19 clusters at immigration detention depots. Malay Mail Online; 2020. Available from: https://www.malaymail.com/news/malaysia/2020/05/25/dr-noor-hisham-confined-spaces-likely-cause-of-covid-19-clusters-at-immigra/1869415. Cited 09 Dec 2021.

[102] Ong JR. Campaign of hate? fake news and anti-refugee rhetoric in Malaysia. New Naratif; 2020. Available from: https://newnaratif.com/campaign-of-hate-fake-news-and-anti-refugee-rhetoric-in-malaysia/. Cited 12 Dec 2021.

[103] Fishbein E. Fear and uncertainty for refugees in Malaysia as xenophobia escalates. The New Humanitarian; 2020. Available from: https://www.thenewhumanitarian.org/news/2020/05/25/Malaysia-coronavirus-refugees-asylum-seekers-xenophobia. Cited 13 Dec 2021.

[104] Office of the High Commissioner for Human Rights. Malaysia / COVID-19: “Stop crackdown on migrants, journalists and civil society” – UN rights experts. OHCHR; 2020. Available from: https://www.ohchr.org/en/press-releases/2020/05/malaysia-covid-19-stop-crackdown-migrants-journalists-and-civil-society-un.

[105] Rashid NZ, Saidin MIS (2023). ‘#SayNoToRohingya’: a critical study on Malaysians’ amplified resentment towards Rohingya refugees on Twitter during the 2020 COVID-19 crisis. Round Table.

[106] Latiff R, Ananthalakshmi A. Anti-migrant sentiment fanned on Facebook in Malaysia. Reuters; 2020. Available from: https://www.reuters.com/article/uk-facebook-malaysia-rohingya-idUKKBN26Z0BP. Cited 13 Dec 2020.

[107] The Straits Times. Malaysia’s health chief warns against discrimination of migrant workers amid coronavirus outbreak at detention centres. The Straits Times; 2020. Available from: https://www.straitstimes.com/asia/se-asia/coronavirus-breaks-out-at-detention-centres-in-malaysia-health-ministry-warns-against. Cited 13 Dec 2021.

[108] Delgado Wise R, Márquez Covarrubias H, Puentes R (2013). Reframing the debate on migration, development and human rights. Popul Space Place.

[109] Rathina Pandi A, Pereira A, Yacob A (2023). Assessment of causes and contributing factors to migrant workers becoming undocumented in Malaysia.

[110] Laczko F (2016). Introduction to the understanding and measuring “safe’’ migration workshop. Understanding and measuring “safe” migration workshop; 21-22 June 2016.

[111] Kaunert C, Leonard S, Wertman O (2022). Securitization of COVID-19 as a security norm: WHO norm entrepreneurship and norm cascading. Soc Sci.

[112] DLA Piper. A global analysis of prisoner releases in response to COVID-19 2020. Available from: https://www.dlapiper.com/~/media/files/insights/publications/2021/03/dla-piper-prison-population-during-covid-19.pdf?la=en&hash=F5C1EBBA0D3D86BDDA58FAC87DB9EF3CAE3815DF. Cited 11 Dec 2021.

[113] FMT. UN warns police raids will push migrants into hiding. Free Malaysia Today Online; 2020. Cited 02 Aug 2023.

[114] von Rosen J (2019). The securitization of migration as a threat to liberal, democratic societies. Sicherheit und Frieden (S+F) / Security and Peace.

[115] SME Corp Malaysia. FAQ 2023. Available from: https://www.smecorp.gov.my/index.php/en/contact-us-2/2015-12-21-11-24-56/faq. Cited 2023 Oct 05.

[116] Yi H, Ng ST, Farwin A, Pei Ting Low A, Chang CM, Lim J (2021). Health equity considerations in COVID-19: geospatial network analysis of the COVID-19 outbreak in the migrant population in Singapore. J Travel Med.

[117] WHO. Health in all policies: Helsinki statement. Framework for country action. Geneva: World Health Organization; 2014. Available from: https://www.who.int/publications/i/item/9789241506908. Cited 2021 Dec 20.

[118] WHO. Valuing health for all: rethinking and building a whole-of-society approach. Council Brief No. 3. Geneva: World Health Organization; 2022. Available from: https://cdn.who.int/media/docs/default-source/council-on-the-economics-of-health-for-all/who_councilbrief3.pdf?sfvrsn=b121f943_11&download=true. Cited 2022 Dec 12.

[119] Willen SS (2012). How is health-related “deservingness” reckoned? Perspectives from unauthorized im/migrants in Tel Aviv. Soc Sci Med.

[120] Shadmi E, Chen Y, Dourado I, Faran-Perach I, Furler J, Hangoma P (2020). Health equity and COVID-19: global perspectives. Int J Equity Health.

